# New concept for treating female stress urinary incontinence with radiofrequency

**DOI:** 10.1590/S1677-5538.IBJU.2016.0621

**Published:** 2017

**Authors:** Patrícia Lordelo, Andrea Vilas Boas, Danielle Sodré, Amanda Lemos, Sibele Tozetto, Cristina Brasil

**Affiliations:** 1 Centro de Atenção ao Piso Pélvico (CAAP), Divisão de Fisioterapeutas e Pós-Graduação, Escola Bahiana de Medicina e Saúde Pública (EBMSP), BA, Brasil; 2 Centro de Atenção ao Piso Pélvico (CAAP), Divisão de Programas de Ginecologia e Pós-Graduação, Escola Bahiana de Medicina e Saúde Pública (EBMSP), BA, Brasil; 3 Divisão de Ciências Morfofuncionais, Universidade Federal do Recôncavo Baiano, BA, Brasil

**Keywords:** Pulsed Radiofrequency Treatment, Urinary Incontinence, Stress, Women

## Abstract

**Purpose:**

To evaluate the clinical response and adverse effects of radiofrequency on the urethral meatus in the treatment of stress urinary incontinence in women.

**Materials and Methods:**

This phase one study included ten women with Stress Urinary Incontinence (SUI). The evaluation consisted of 1 hour Pad tests to quantify urine loss and to assess the degree of procedure satisfaction by using the Likert scale. To evaluate safety, we observed the number of referred side effects.

**Results:**

Average age was 53.10 years±7.08 years. In assessing the final Pad Test, 70% showed a reduction and 30% a worsening of urinary loss. Using the Pad Test one month later, there was a reduction in all patients (p=0.028). The degree of satisfaction was 90% and no side effects have been observed. One patient reported burning sensation.

**Conclusion:**

The treatment of SUI with radiofrequency on the urethral meatus has no adverse effects, being a low risk method that reduces urinary loss in women. However, to increase the validity of the study, larger clinical trials are warranted.

## INTRODUCTION

The prevalence of stress urinary incontinence (SUI) in the adult female population varies widely, ranging from 4% to 35% (1, 2). SUI has a high impact on the health condition of patients, with personal and social consequences and high negative impact on psychological and relational well-being (3, 4). This justifies a continuous search for a therapy.

The first line of treatment is pelvic floor muscle training. Medication, or even surgery, could also be recommended. The success rate of therapeutic treatment varies from 25 to 90%, depending on severity, cause and timing of reassessment (5).

Theetiology of SUI is multifactorial, and SUI may be caused by inadequate support of the pelvic organs and anterior vaginal wall suspension and/or a possible change in the intrinsic urethral closure mechanism itself (6, 7). In addition to that, histological studies observed a reduction of collagen in urethra walls in case of loss of urethral support and/or sphincter dysfunction (8), making therapy with radiofrequency an option.

A current treatment proposal is the use of radiofrequency, which is a diathermic process generated by the radiation of an electromagnetic spectrum, resulting in an immediate retraction of existing collagen and subsequent activation of fibroblasts causing neocollagenesis (9). In studies using radiofrequency to treat SUI, a therapeutic response of 50% was shown (10). Elser et al., used the probe by inserting it in the intra urethral or intravaginal region. Although this technique is minimally invasive, it presented a rate of adverse or side effect of 0.9% to 9.5%, and the need for antibiotic prophylaxis, oral sedation, local anesthesia, while increasing the risk of urinary tract infections and its costs (11, 12).

Female urethra is known for having a maximum length of five centimeters, and its anatomical structure and length justifies the use of radiofrequency on the external urethral meatus. Radiofrequency waves can reach a sufficient depth to induce collagen production in the whole urethra. The hypothesis of this innovative study is that radiofrequency treatment on the urethral meatus reduces urinary loss, in a safe manner and with low risk. Our main objective is to evaluate the clinical response and adverse effects of radiofrequency on the urethral meatus in the treatment of stress urinary incontinence in women.

## MATERIALS AND METHODS

These are results of a phase one study, approved by the Ethics Committee and Research of the Bahia School of Medicine and Health Public (CAAE: 20333213.1.0000.5544). Conformed to the standards set by the Declaration of Helsinki. It was registered at ClinicalTrials.gov (NTR: 02623842). All participants provided written informed consent.

The age of the women involved varied from 43 to 66 years, with an average of 53.1±7.1 years. Eligible women were at least 18 years of age, with SUI as the main clinical complaint, without any urgency symptoms (clinical complaint plus voiding diary per three days), and urinary loss of more than 1g in a one hour Pad Test. Patients with organ prolapses, neurological chronic degenerative diseases, residual voiding, pacemakers, copper intrauterine devices, or those who underwent other treatment for SUI (medical, surgical or physical therapy) as well as pregnant women, were excluded.

### Evaluation of patients

Initially an anamnesis questionnaire was carried out to assess the presence of comorbidities, associated urinary symptoms and fecal urinary symptoms. After the questionnaire was done, physical examination took place to assess the function of the muscles of the pelvic floor. The examination comprised digital palpation quantified by the modified Oxford scale (13).

### Device and procedure description

**Figure 1:**
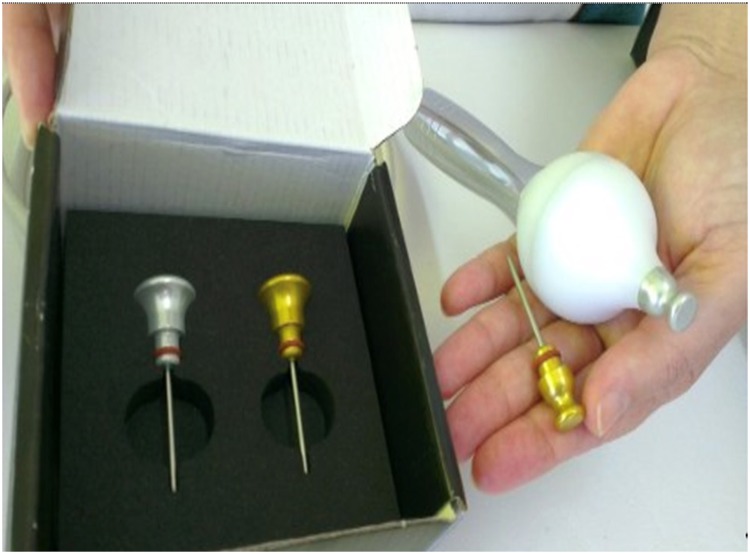
Details of the electrodes used in the radiowfrequency apparatus Spectra G3 Tonederm^®^.

The non-ablative radio frequency device Spectra G2 - Tonederm^®^, has been adjusted for use on the urethral meatus. The device consists of an electromagnetic wave generator - high frequency wave, 0.5MHz - which is connected to a monopolar active electrode with a diameter of 0.5cm, and a passive metal electrode, the return plate (Figure-1). Only equipment with the approval of the national organ (ANVISA) can be used for this treatment method.

The undressed patient lays in lithotomy position, the return plate is placed under the sacrum and the active electrode is positioned on the external urethral meatus.

**Figure 2:**
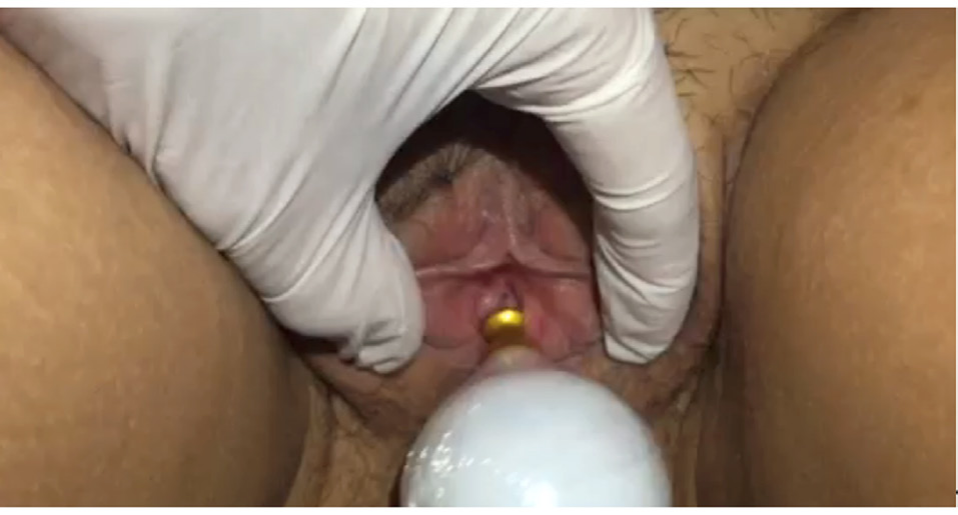
Demonstration of application of non-ablative radiofrequency in external urethral meatus.

When starting the passage of electromagnetic waves, the active electrode is placed on the urethral meatus and moved in circles (Figure-2). The active electrode is removed regularly to perform the temperature check. The temperature is monitored with an infrared thermometer, and after reaching 39-41°C, this temperature and the motions are maintained for 2 minutes. We used the same principle of the monopolar radiofrequency that is used for tissue repair to genital regions (14). All patients had 5 sessions of treatment, with a weekly frequency, and did not undergo any other therapeutic treatment for urinary incontinence. Women currently taking medications such as hormones, diuretics, or other medications, remained on their usual dose of medicine throughout the study period.

### Assessment of response to therapy

As objective evaluation, the Pad test was repeated immediately after the last radiofrequency treatment, and as follow-up one-, two- and three months after the treatment. The categories used in the classification of urinary loss are: loss of 1 to 10g represents mild incontinence, 11 to 50g represents moderate incontinence and >50g represents severe incontinence (3).

As subjective evaluation, the level of patient satisfaction was measured using a 5-point Likert scale, which measured the response to treatment as follows: 1) very dissatisfied, 2) dissatisfied 3) neutral; 4) satisfied; 5) very satisfied.

The expected adverse effects were edema, redness, increased local temperature or presence of secretion.

### Statistical Design

To prepare the database and descriptive analysis, the Statistical Package for Social Sciences software (SPSS Inc., Chicago, IL, USA) version 14.0 for Windows, was used. The results are presented in tables and graphs. Categorical variables (patient satisfaction level) are expressed as frequencies and percentages -n (%). Continuous variables with normal distribution are expressed as mean and standard deviation; and those with non-normal distribution, as median and interquartile range. The normality of the numerical variables was assessed using descriptive statistics, graphical analysis and the Shapiro -wilk test.

The analysis of the mean Pad Test comparison was performed by ANOVA repeated measures, and compared the loss in grams at the begginning, the end, after one-, two-, and three months of treatment, considering a significance level of 5% (p <0.05).

## RESULTS

The sample consisted of 10 patients with a mean age of 53.10±7.08 years. The clinical characteristics are shown in Table-1. The result of the initial Pad test evaluation showed four (40%) participants classified as having experienced a slight loss, five (50%) a moderate loss and one (10%) a severe loss.

**Table 1:** Clinical characteristics of 10 patients who underwent non-ablative radiofrequency treatment on the external urethral meatus, Salvador - BA, 2015.

Patient	Age	Pelvic floor muscle strength*	Pregnancies	Normal deliveries	Surgeries	Medicins for CNS and LUTS	Hormonal status	Smoking
01	56	4	0	0	Hemorrhoidectomy	HRT	Menopause	No
02	62	1	5	5	TAH	HRT	Menopause	No
03	49	4	4	4	No	High blood pressure Clordalidone Elanapril	Fertile	No
04	43	3	2	1	Myomectomy	Captopril pressure Puran t4	Fertile	No
05	47	4	2	0	Caesarean section	OC, Nifedipine, Hydrochlorothiazide	Fertile	No
06	49	3	3	3	TAH	No	Fertile	No
07	51	2	6	4	No	No	Menopause	No
08	57	1	7	5	No	No	Menopause	Yes
09	66	1	8	4	TAH	Losartan, Hydrochlorothiazide	Menopause	No
10	51	4	2	1	TAH + Perineoplasty	Estradot + Testosterone	Menopause	Yes

**CNS =** Central Nervous System; **AO =** Anticoncepcional Oral; **OC =** Oral Contraceptive; **LUT =** Lower Urinary Tract; **TAH =** Total Abdominal Hysterectomy

* measure by modified Oxford scale.

In assessing the final Pad test, seven (70%) showed a reduction of urinary loss, two (20%) showed no further loss and three (30%) a worsening of urinary loss.

**Figure 3:**
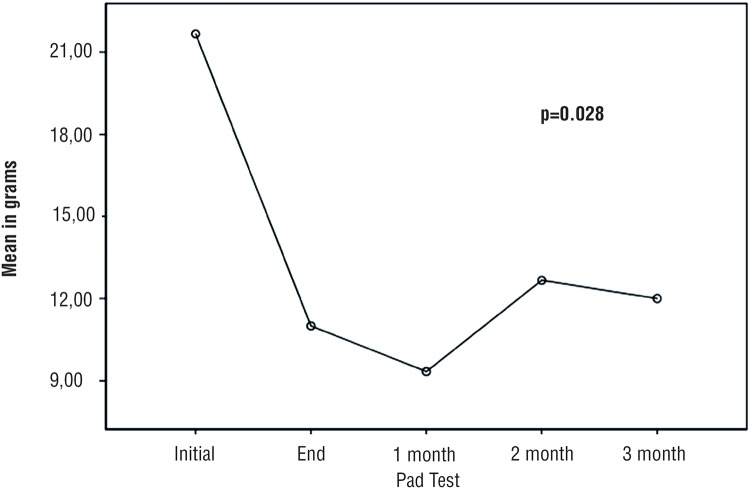
Comparison of mean urinary loss in grams (g) Pad Test at the begginning-, end-, and after one-, two- and three months of treatment.

Figure-3 shows reduction of the urine loss in grams (g) Pad test at the begginning-, end-, and after one-, two - and three months of treatment (p=0,028).

After one month, all participants showed an improvement in the results of Pad test, compared to the initial examination: two (20%) had no loss, three (30%) had a slight loss, four (40%) had a moderate loss and none had severe loss. One participant did not return for a follow-up review after one month (Table-2).

**Table 2:** Results of urinary loss in grams (g) of 10 patients who underwent non-ablative radiofrequency treatment on external urethral meatus measured by Pad Test, Bahia, 2015.

Patient	Initial Pad test (g)	Final (End) Pad test (g)	Pad test after 1 month (g)	Pad test after 2 months (g)	Pad test after 3 months (g)
01	2	1	0	2	2
02	6	2	0	5	4
03	13	23	10	12	--
04	16	21	10	31	29
05	7	2	5	3	--
06	6	3	5	11	--
07	11	10	4	3	6
08	25	27	20	19	16
09	70	5	22	16	15
10	16	0	--	2	--

While assessing patient satisfaction, nine (90%) participants reported to be satisfied with the treatment. One patient indicated to be little satisfied with the treatment as an answer to the Likert questionnaire.

Assessing whether the use of non-ablative radiofrequency on urethral meatus is regarded safe, nine (90%) out of 10 participants presented no adverse or side effects. One participant indicated to have felt an unexpected burning sensation in the area of the urethral meatus, just after the menstrual period. This participant returned for the radiofrequency treatment a week later, without any complaints.

During her physical examination, there was no edema, redness, increased local temperature or presence of secretion. Nothing was prescribed in order to improve this discomfort. No other complications were observed. All patients completed the five sessions.

## DISCUSSION

We present a new technique of a conservative treatment of SUI. It follows a principle that already exists, but applied in a different way. We applied the technique on the urethral meatus after an animal experimental study has demonstrated the possibility of increasing collagen production of anal sphincter (9) and we used temperatures ranging from 39 to 41°C, as it was proved to be safe and effective in the human genital region (14).

We demonstrated that the method is painless and reliable. Besides the fact that we indicate its lower risk of adverse effects during the technical treatment; only one patient reported a burning sensation during a session right after menstruation. In this case, a possible difference in resistance of tissue could be due to friction of the pad with a change in impedance of the passage of electric waves. For the electric current to perform the desired action on the tissue, it needs to overcome the barrier imposed on its flow and reach the target tissue in the right intensity. This is what we call tissue impedance. The impedance is composed of the extra flow resistance and capacitive reactance of cell membranes. The electric current will always take the path of least resistance. The tissue impedance may change the density, intensity and path of the current and of the biological response (15).

The method has no adverse effects; the observed results were similar to those of the studies of Meillheiser et al. where radiofrequency was used for treatment of the vaginal introitus to treat vaginal laxity; and a pilot study conducted to test tolerance and safety showed that there has been no adverse effect (using frequency 75-90 Joules/cm^2^) (16). In addition to the low risk, one advantage of this new treatment technique is that it is not necessary to place the device in the urethra, which reduces the side effects and eliminates the need for prophylactic antibiotics or the use of anesthetics, as used in prior studies. In the systematic review on the intraurethral radiofrequency technique, a relative risk (RR) of 5.76 of pain / burning-, a RR of 1.36 of a hyperactive destrusor-, and a RR of 0.95 of urinary retention was found (17).

The clinical response related to urinary loss was satisfactory for this group of patients studied. This is a very small number of patients to show therapeutic effectiveness, but considered a necessary phase study when to present a new therapy. Randomized clinical trials are being developed by our group to assess the effectiveness of the method.

The improvement in urinary loss is shown in the final Pad test. Seven out of ten participants showed an improvement in reducing stress urinary incontinence. By treating with radiofrequency, local temperatures increase, which enables vasodilation and the opening of capillaries, the gain of oxygen, and an improved drainage. This phenomenon can improve the circulation of the venous plexus which is a layer of spongy erectile tissue, that contributes to the urethral closure mechanism (7). The decrease in urinary loss measured by the Pad test was most evident one month after the radiofrequency treatment. The result we found is probably due to the period of collagen denaturation and neo production that remains until 28 days after treatment. Since those collagen changes favor the urethral closure mechanism (18), it could be explained why there was a better response on the pad test after one month. Rechberger et al. demonstrated that collagen content has been correlated with the urethral pressure, the length of the urethra and maximum closure pressure of the urethra (19).

Nine out of ten patients indicated to be satisfied with the treatment, although seven out of ten patients experienced a reduction of urinary loss after treatment with radiofrequency. This finding shows that satisfaction is not only linked to the therapeutic outcome, but possibly also to the level of expectations of the people involved. This means that degrees of satisfaction do not always correspond to the results. However, the degree of satisfaction should be measured to stablish a subjective response of patients. Satisfaction is the feeling of pleasure or disappointment which resulted from comparing a perceived performance or outcome against one's expectations. When considering the answers of patients on satisfaction, the Hawthorne-effect can be taken into consideration. Hawthorne said that when individuals believe they are experiencing a form of treatment, they are more likely to respond to be satisfied with therapeutic responses (20). Another factor that should be taken into consideration is that the complaint regarding urinary loss is not directly proportional to the volume of urine loss (21, 22).

A disadvantage of this new technique is that qualified professionals are required to perform the procedures. Another issue is a need to schedule five sessions, however, there is no consensus on parameters and treatment frequency in literature regarding radiofrequency treatment.

Hence, it can be considered a conceivable perspective to carry out a clinical trial to measure the response to radiofrequency treatment on the external urethral meatus of woman with SUI, with a control on variables such as age, parity, degree of muscle strength, BMI, and with a long-term control of the response to therapy on the external urethral meatus. A limitation found was the loss of 4 patients during the follow-up phase, due to their lack of finances to finish the study.

## CONCLUSIONS

The preliminary results of our study (phase 1) look promising. However, to increase the validity of the study, larger clinical trials are warranted. Our study showed that the treatment of stress urinary incontinence with radiofrequency on the urethral meatus had no adverse effects and reduced urinary loss in women.
